# Low handgrip strength is closely associated with chronic low back pain among women aged 50 years or older: A cross-sectional study using a national health survey

**DOI:** 10.1371/journal.pone.0207759

**Published:** 2018-11-26

**Authors:** Sang-Min Park, Gang-Un Kim, Ho-Joong Kim, Hyoungmin Kim, Bong-Soon Chang, Choon-Ki Lee, Jin S. Yeom

**Affiliations:** 1 Spine Center and Department of Orthopaedic Surgery, Seoul National University College of Medicine and Seoul National University Bundang Hospital, Seongnam, Republic of Korea; 2 Department of Orthopedic Surgery, Sanggye Paik Hospital, College of Medicine, Inje University, Seoul, Republic of Korea; 3 Department of Orthopaedic Surgery, Seoul National University College of Medicine and Seoul National University Hospital, Seoul, Korea; Universidad Autonoma de Queretaro, MEXICO

## Abstract

**Object:**

This study aimed to analyze the association between low handgrip strength (HGS) and low back pain (LBP) according to physical activity (PA) in the general population aged over 50 years.

**Methods:**

Nationwide health surveys and examinations were performed in a cross-sectional representative of the Korean general population (n = 7,550 in 2014, n = 7,380 in 2015). Chronic LBP status was determined by self-reported survey responses with respect to the occurrence of LBP for more than 30 days during the previous 3 months. Maximal HGS was determined as the maximal strength of the dominant hand, and low HGS was defined as measurement in the lower 20th percentile of HGS measurements for the general population. High PA was defined as muscle-strengthening exercise for at least 3 days within 1 week. Demographics, medical history, and other variables were used to analyze adjusted weighted logistic regression models with propensity score matching. After propensity score matching, 429 participants were included in each group.

**Results:**

Analysis was confined to those aged 50–89 years who responded to the chronic LBP survey and had no missing data on HGS. Low HGS and LBP showed significant association in the crude logistic regression model. In the multiple logistic regression model, after adjusting for confounding factors, low HGS was significantly associated with LBP in women with low PA (adjusted odds ratio [aOR]: 1.75, *p* = 0.047). In the logistic regression model after propensity score matching, low HGS was also significantly related to LBP in women with low PA (aOR: 3.12, *p* = 0.004).

**Conclusions:**

Our study showed the relationship between low HGS and LBP using a cross-sectional Korean population-based health survey. Low HGS in women aged over 50 years with low PA was significantly associated with the presence of LBP.

## Introduction

Low back pain (LBP) is the most common musculoskeletal problem affecting quality of life and function in the elderly worldwide and is experienced by approximately 70% of people in their lifetime [1][2][3]. The etiologies of LBP not only are multifactorial and complex but also remain poorly understood. LBP is directly related to anatomical problems in the spine, such as disc degeneration, stenosis, or sprain; however, other physical or environmental factors also exist. Most studies have reported a reduction in LBP with increased physical activity, indicating a relationship between muscle strength and LBP [4][5][6]. According to one study that analyzed the direct relationship between trunk muscle strength and LBP, isometric and isokinetic extensor weakness was directly associated with LBP [7]. Similar to this study, one study showed that walking speed, which is one of the diagnostic criteria for sarcopenia, was negatively correlated to Oswestry disability index (ODI) score [8]. As opposed to these studies, one study reported no relationship between LBP and total or appendicular muscle mass, which is also one of the diagnostic criteria for sarcopenia [9]. To date, no study has investigated the relationship between muscle strength and LBP in the general population.

Handgrip strength (HGS) is a simple and reliable measurement technique for the assessment of maximal voluntary hand force [10]. HGS is a useful tool for measuring general muscle strength to diagnose sarcopenia, as low HGS is a clinical indicator of poor mobility, low muscle mass, and poor nutritional status [11][12]. We hypothesized that low muscle strength is related to LBP, and we evaluated this relationship using HGS instead of trunk muscle strength.

Based on these theoretical considerations, this study aimed to analyze the association between LBP and HGS according to physical activity, which represents muscle strength, in a general population aged 50 years and older using a representative community sample.

## Materials and methods

### Study participants

**Fig 1:**
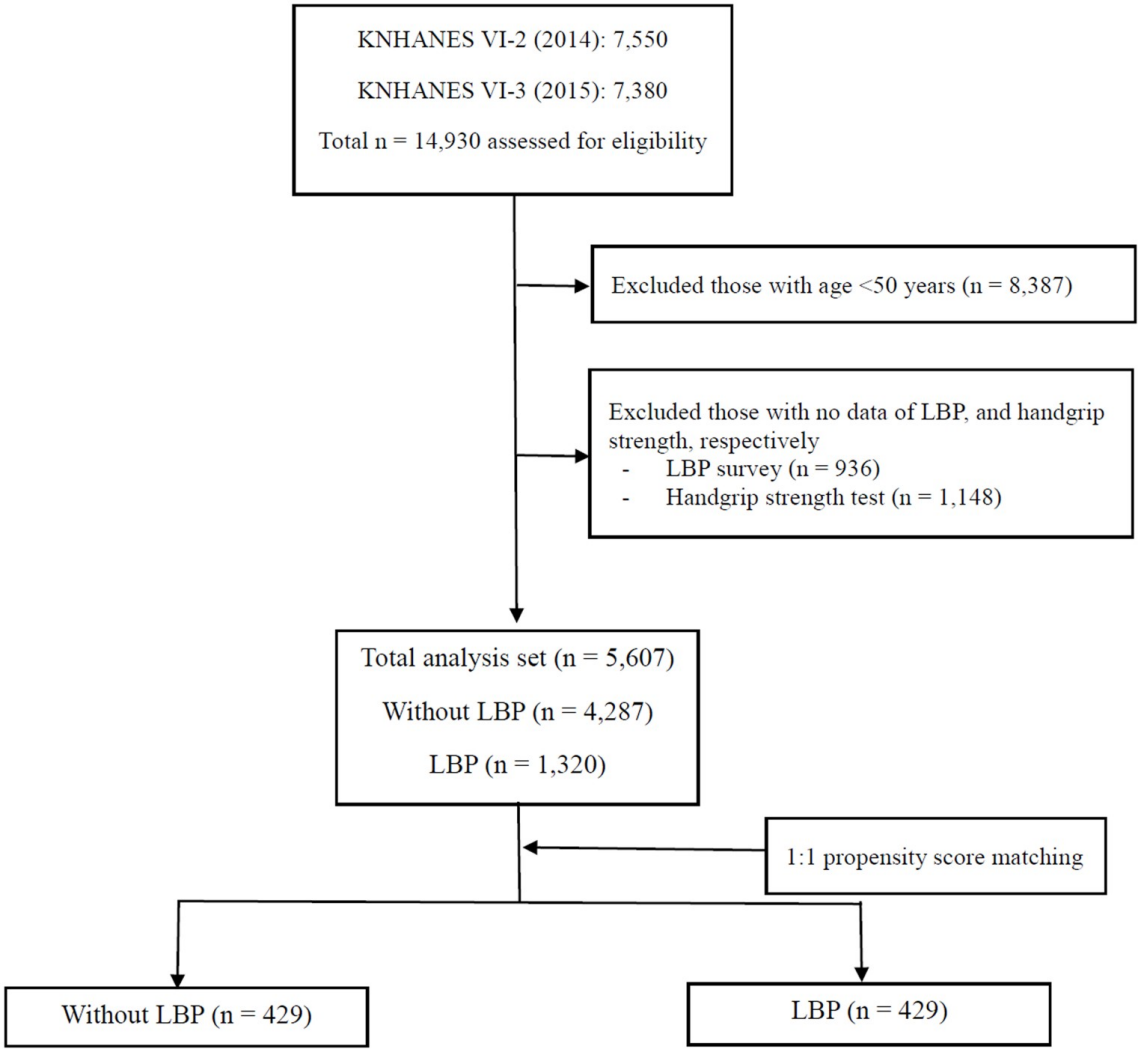
Flow diagram of inclusion and exclusion of participants from the 2014 and 2015 Korea National Health and Nutrition Examination Surveys (KNHANES VI-2 and VI-3). LBP, low back pain.

The Korea National Health and Nutrition Examination Survey (KNHANES) versions V-2 and V-3 were performed in 2014 and 2015, respectively. This survey has been annually conducted since 1998 by the Korea Centers for Disease Control and Prevention (KCDC) to evaluate the health and nutritional status of the Korean general population using a nationwide, clustered, multistage, stratified, and randomized sampling method that is proportionally distributed according to geographic area, sex, and age. The survey participants are different every year and are not serially monitored, resulting in annual random sampling. The KNHANES evaluates three aspects: health surveys, health examinations, and dietary questionnaires that are administered by experienced interviewers, registered nurses, and laboratory technicians [13]. Health surveys and examinations were completed by 7550 participants in KNHANES V-2 (2014) and 7580 participants in KNHANES V-3 (2015). Among 14,930 participants in the 2014 and 2015 KNHANES surveys, a total of 5607 participants who completed the LBP questionnaire and the HGS test were included in this study. Of these, 4287 participants had no LBP (non-LBP), whereas 1320 participants reported LBP. After propensity score matching (refer to “Statistical analysis” section), 429 participants were included in each group (Fig 1).

The KNHANES VI-2 and VI-3 were approved by the KCDC Institutional Review Board (approval no. 2013-12EXP-03-5C). Informed consent was obtained from all participants when the surveys were conducted.

### Definitions of low back pain and physical activity

In our study, LBP was defined as individuals who answered “yes” to the question “Have you complained of LBP for more than 30 days during the past 3 months?” This LBP definition includes all types of LBP, such as disc herniation, stenosis, tumor, trauma, and non-specific LBP. High physical activity was defined as muscle-strengthening exercise, such as push-ups, sit-ups, and dumbbell curls, for at least 3 days within 1 week.

### Measurement of handgrip strength

HGS was measured three times in each hand using a digital handgrip dynamometer (TKK 5401, Takei Scientific Instruments Co., Ltd., Tokyo, Japan). Trained nurses taught each participant to grip the dynamometer with the second finger node at 90° angle to the handle and to grab the handle as strongly as the participant could. Maximal grip strength was checked with the forearm away from the body in standing position. There were intervals of least 30 s between three HGS measurements for each hand. Maximal HGS was defined as the highest value of the six measurements.

HGS was divided into two categories based on a previous study that defined HGS reference values for low muscle strength [11]. Therefore, in accordance with the HGS cut-off values in the previous study, low muscle strength was defined in this study as grip strength <28.6 kgf and <16.4 kgf in men and women, respectively.

### Characteristics of the study population

The participants provided data on their demographics, socioeconomic characteristics, medical history (e.g., hypertension, diabetes), nutritional status, and other characteristics in the health and nutritional surveys and examinations conducted by interviewers. We only used the raw data from the health surveys and examinations, from which we extracted items related to LBP, as described below.

Basic characteristics, such as age, sex, and body mass index, were described and analyzed. Smoking status was categorized into nonsmoker/ex-smoker and current smoker. Alcohol consumption status was categorized as follows: none, ≤1 drink/month, 2 drinks/month to 3 drinks/week, and ≥4 drinks/week. Current occupational status was categorized into the following five groups: unemployed (e.g., student, homemaker); office worker (e.g., manager, professional); sales and services, machine fitting, and simple labor (e.g., technician, device and machine operator, and low-level laborer); and agriculture, forestry, and fishery [14]. Household income level was categorized into quartiles. Educational level was divided into the following four groups: ≤6 years, 7–9 years, 10–12 years, and ≥13 years. The medical history of participants was assessed as to whether they had been diagnosed with major comorbidities such as hypertension, diabetes mellitus, chronic kidney disease, dyslipidemia, ischemic heart disease (myocardial infarction, angina), stroke, liver cirrhosis, chronic hepatitis B or C, major cancers (lung, stomach, liver, colon, breast, or uterine cervical), asthma, pulmonary tuberculosis, depression, or arthritis. We also counted the number of major comorbidities and divided them into three groups.

### Statistical analysis

Statistical analyses were performed using Stata/MP 15.0 (Stata Statistical Software: Release 15; StataCorp LP, College Station, TX, USA). To reduce the baseline difference in comorbidities, we used a propensity score matching technique. A logistic regression model with the following selected variables was used: age, sex, body mass index, smoking status, alcohol consumption, current occupation, household income, educational level, and number of major comorbidities. Propensity score matching was subsequently performed with 1:1 matching using the nearest neighbor matching method without replacement. We used the “pscore” and “psmatch2” modules of Stata to calculate the propensity score. The adequacy of the model was assessed using standardized bias (%) that was tested with the “pstest” module of Stata [15].

With respect to the characteristics of the study population with LBP and without LBP after propensity score matching according to sex, Student’s t-test was used for continuous variables, whereas the chi-squared test was used for categorical variables. To ensure that differences due to confounding factors were not attributed to low HGS, logistic regression analyses were performed using three different models. First, we analyzed the odds ratios (ORs) in the study population before propensity score matching (crude model) by logistic regression. Afterwards, multiple logistic regression analyses adjusted for confounding factors were performed, and propensity score matching was also carried out to calculate the ORs in the study population (before propensity score matching) (model 1). A third logistic regression analysis was performed in the matched population after propensity score matching (model 2). ORs with corresponding 95% confidence intervals were accordingly calculated. Sampling weights were applied to the study population to represent the Korean population without bias. All statistics were two-tailed, and *p* < 0.05 was considered statistically significant.

## Results

### Characteristics of participants according to low back pain

The clinical characteristics of the study population based on propensity score matching are summarized in Table 1. According to standardized bias (%), the clinical characteristics of non-LBP and LBP groups were biased before propensity score matching. This was significantly reduced after propensity score matching, although small differences remained. A total of 858 participants after propensity score matching were divided according to sex (Table 2). In men (n = 294), most characteristics including HGS were not significantly different between groups, except for age, current occupation, and household income. Although a similar pattern for characteristics was observed in women (n = 564), HGS was significantly different between non-LBP and LBP groups (*p* = 0.002).

**Table 1:** Characteristics of the study population according to low back pain before and after propensity score matching.

Characteristics	Before propensity score matching			After propensity score matching		
	Without LBP (n = 4,287)	LBP (n = 1,320)	Standardized bias ^a^ (%)	Without LBP (n = 429)	LBP (n = 429)	Standardized bias (%)
Age, n (%)			40.7			0.8
50–59	1680 (39.2%)	339 (25.7%)		168 (39.2%)	117 (27.3%)	
60–69	1467 (34.2%)	371 (28.1%)		145 (33.8%)	132 (30.8%)	
70–79	934 (21.8%)	470 (35.6%)		94 (21.9%)	147 (34.3%)	
≥80	206 (4.8%)	140 (10.6%)		22 (5.1%)	33 (7.7%)	
Sex, n (%)			45.8			-1.0
Male	2045 (47.7%)	341 (25.8%)		185 (43.1%)	109 (25.4%)	
Female	2242 (52.3%)	979 (74.2%)		244 (56.9%)	320 (74.6%)	
BMI, kg/m^2^	24.1 (3.1)	24.4 (3.4)		24.0 (3.4)	24.1 (3.2)	
Smoking status, n (%)			-6.4			2.9
Non / Ex-smoker	3582 (85.3%)	1117 (88.7%)		362 (84.4%)	372 (86.7%)	
Current smoker	619 (14.7%)	143 (11.3%)		67 (15.6%)	57 (13.3%)	
Alcohol consumption, n (%)			-18.3			-0.6
None	823 (23.6%)	270 (29.1%)		83 (22.9%)	92 (28.1%)	
≤1 drink/month	1050 (30.1%)	334 (36.0%)		126 (34.8%)	120 (36.7%)	
2 drinks/month to 3 drinks/week	1245 (35.6%)	244 (26.3%)		119 (32.9%)	87 (26.6%)	
≥4 drinks/week	375 (10.7%)	81 (8.7%)		34 (9.4%)	28 (8.6%)	
Occupation, n (%)			12.7			-2.4
Unemployed (Student, housewife, etc.)	1961 (46.1%)	798 (61.6%)		194 (45.2%)	280 (65.3%)	
Office work	477 (11.2%)	63 (4.9%)		39 (9.1%)	20 (4.7%)	
Sales and services	483 (11.4%)	109 (8.4%)		55 (12.8%)	35 (8.2%)	
Agriculture, forestry and fishery	365 (8.6%)	123 (9.5%)		37 (8.6%)	24 (5.6%)	
Machine fitting and simple labor	965 (22.7%)	202 (15.6%)		104 (24.2%)	70 (16.3%)	
Household income, n (%)^b^			-44.2			-0.9
Low	1038 (24.3%)	597 (45.4%)		100 (23.3%)	181 (42.2%)	
Low-moderate	1183 (27.7%)	324 (24.6%)		114 (26.6%)	111 (25.9%)	
Moderate-high	1000 (23.4%)	206 (15.7%)		101 (23.5%)	72 (16.8%)	
High	1045 (24.5%)	188 (14.3%)		114 (26.6%)	65 (15.2%)	
Educational level, n (%)			-50.8			-0.1
≤6 years	1559 (36.7%)	793 (61.3%)		152 (35.4%)	244 (56.9%)	
7–9 years	771 (18.1%)	182 (14.1%)		87 (20.3%)	70 (16.3%)	
10–12 years	1166 (27.4%)	214 (16.5%)		129 (30.1%)	76 (17.7%)	
≥13 years	754 (17.7%)	105 (8.1%)		61 (14.2%)	39 (9.1%)	
Comorbidities, n (%)^c^			46.0			0.0
0	1597 (37.3%)	272 (20.6%)		163 (38.0%)	91 (21.2%)	
1	1332 (31.1%)	371 (28.1%)		129 (30.1%)	119 (27.7%)	
≥ 2	1358 (31.7%)	677 (51.3%)		137 (31.9%)	219 (51.0%)	

Numeric parameters are expressed as mean and standard deviation in parentheses

Categorical parameters are expressed as counts and percentages in parentheses

LBP; low back pain, BMI; body mass index

^a^ The adequacy of the model was assessed using standardized bias (%) which tested by “pstest” module of STATA.

^b^ Household income level was calculated by dividing the total household monthly income with the obtained levels then grouped into quartiles

^c^ Number of major comorbidities: hypertension, diabetes mellitus, dyslipidemia, ischemic heart disease (myocardial infarction, angina), stroke, liver cirrhosis, major cancers (lung, stomach, liver, colon, breast, or uterine cervical), asthma, pulmonary tuberculosis, arthritis, or chronic kidney disease

**Table 2:** Characteristics of the study population after propensity score matching according to gender.

Characteristics	Male			Female		
	Without LBP (n = 185)	LBP (n = 109)	p-value	Without LBP (n = 244)	LBP (n = 320)	p-value
Age, n (%)			0.016			0.003
50–59	66 (35.7%)	25 (22.9%)		102 (41.8%)	92 (28.7%)	
60–69	71 (38.4%)	37 (33.9%)		74 (30.3%)	95 (29.7%)	
70–79	42 (22.7%)	42 (38.5%)		52 (21.3%)	105 (32.8%)	
≥80	6 (3.2%)	5 (4.6%)		16 (6.6%)	28 (8.8%)	
BMI, kg/m^2^	23.9 (3.1)	23.5 (2.9)	0.36	24.2 (3.6)	24.3 (3.2)	0.60
Smoking status, n (%)			0.19			0.51
Non / Ex-smoker	126 (68.1%)	66 (60.6%)		236 (96.7%)	306 (95.6%)	
Current smoker	59 (31.9%)	43 (39.4%)		8 (3.3%)	14 (4.4%)	
Alcohol consumption, n (%)			0.76			0.48
None	35 (19.8%)	19 (18.8%)		48 (25.9%)	73 (32.3%)	
≤1 drink/month	38 (21.5%)	18 (17.8%)		88 (47.6%)	102 (45.1%)	
2 drinks/month to 3 drinks/week	75 (42.4%)	43 (42.6%)		44 (23.8%)	44 (19.5%)	
≥4 drinks/week	29 (16.4%)	21 (20.8%)		5 (2.7%)	7 (3.1%)	
Occupation, n (%)			0.005			0.005
Unemployed (Student, housewife, etc.)	58 (31.4%)	58 (53.2%)		136 (55.7%)	222 (69.4%)	
Office work	24 (13.0%)	9 (8.3%)		15 (6.1%)	11 (3.4%)	
Sales and services	12 (6.5%)	6 (5.5%)		43 (17.6%)	29 (9.1%)	
Agriculture, forestry and fishery	24 (13.0%)	6 (5.5%)		13 (5.3%)	18 (5.6%)	
Machine fitting and simple labor	67 (36.2%)	30 (27.5%)		37 (15.2%)	40 (12.5%)	
Household income, n (%)^a^			0.006			<0.001
Low	45 (24.3%)	44 (40.4%)		55 (22.5%)	137 (42.8%)	
Low-moderate	45 (24.3%)	27 (24.8%)		69 (28.3%)	84 (26.3%)	
Moderate-high	47 (25.4%)	25 (22.9%)		54 (22.1%)	47 (14.7%)	
High	48 (25.9%)	13 (11.9%)		66 (27.0%)	52 (16.3%)	
Educational level, n (%)			0.17			<0.001
≤6 years	53 (28.6%)	45 (41.3%)		99 (40.6%)	199 (62.2%)	
7–9 years	37 (20.0%)	17 (15.6%)		50 (20.5%)	53 (16.6%)	
10–12 years	56 (30.3%)	27 (24.8%)		73 (29.9%)	49 (15.3%)	
≥13 years	39 (21.1%)	20 (18.3%)		22 (9.0%)	19 (5.9%)	
Comorbidities, n (%)^b^			0.27			<0.001
0	73 (39.5%)	33 (30.3%)		90 (36.9%)	58 (18.1%)	
1	56 (30.3%)	36 (33.0%)		73 (29.9%)	83 (25.9%)	
≥ 2	56 (30.3%)	40 (36.7%)		81 (33.2%)	179 (55.9%)	
Hand grip strength ^c^			0.36			0.002
Low muscle strength	20 (11.3%)	14 (15.2%)		10 (4.4%)	31 (12.3%)	
Normal muscle strength	157 (88.7%)	78 (84.8%)		215 (95.6%)	221 (87.7%)	

Numeric parameters are expressed as mean and standard deviation in parentheses

Categorical parameters are expressed as counts and percentages in parentheses

LBP; low back pain, BMI; body mass index

^a^ Household income level was calculated by dividing the total household monthly income with the obtained levels then grouped into quartiles

^b^ Number of major comorbidities: hypertension, diabetes mellitus, dyslipidemia, ischemic heart disease (myocardial infarction, angina), stroke, liver cirrhosis, major cancers (lung, stomach, liver, colon, breast, or uterine cervical), asthma, pulmonary tuberculosis, arthritis, or chronic kidney disease

^c^ Hand grip strength was divided into 2 categories according to previous study which defined reference values of HGS for determining low muscle strength. The cut-off values of Hand grip strength in men and women were 28.6 kgf and 16.4 kgf, respectively. According to these values, we defined low muscle strength as below 28.6 kgf in men and 16.4 kgf in women.

### Relationship between low handgrip strength and low back pain according to physical activity

The association between HGS and LBP was investigated (Table 3). Univariate multiple regression analyses showed that the presence of low HGS was significantly associated with LBP (crude model; OR = 2.30, *p* < 0.001). It also applied equally to men and women (OR = 2.42, *p* < 0.001; OR = 2.39, *p* < 0.001). In model 1 adjusted for several confounding factors, the presence of low HGS was associated with LBP in the low physical activity group (OR = 1.36, *p* = 0.044). However, in the subgroup analysis according to sex, statistical significance was only observed in women (OR = 1.75, *p* = 0.047). In model 2, which was analyzed in matched groups, it was again noted that low HGS was associated with LBP, especially in women with low physical activity (OR = 3.12, *p* = 0.004).

**Table 3:** Association between low hand grip strength and low back pain using multiple logistic regression and propensity score-matched analysis.

	Overall			Male			Female		
	OR	95% CI	*P*	OR	95% CI	*P*	OR	95% CI	*P*
Crude ^a^									
Normal muscle strength	1			1			1		
Low muscle strength	2.30	1.75–3.02	< 0.001	2.42	1.63–3.59	< 0.001	2.39	1.69–3.39	< 0.001
Model 1^b^									
Low physical activity									
Normal muscle strength	1			1			1		
Low muscle strength	1.36	1.01–1.84	0.044	1.54	0.949–2.52	0.080	1.75	1.01–3.04	0.047
High physical activity ^c^									
Normal muscle strength	1			1			1		
Low muscle strength	0.81	0.22–2.87	0.746	0.64	0.14–2.93	0.567	0.89	0.12–6.39	0.907
Model 2^d^									
Low physical activity									
Normal muscle strength	1			1			1		
Low muscle strength	1.89	1.13–3.17	0.015	1.38	0.62–3.07	0.432	3.12	1.45–6.75	0.004
High physical activity									
Normal muscle strength	1			1			1		
Low muscle strength	1.40	0.30–6.67	0.670	1.50	0.23–9.87	0.673	1.47	0.08–25.32	0.792

OR, Odds ratio; 95% CI, 95% confidence interval.

^a^ Crude was unadjusted odds ratio before propensity score matching

^b^ Model 1 was calculated by multiple logistic regression adjusted for age, gender, body mass index, smoking status, alcohol consumption, occupation, household income, education level, and the number of major comorbidities. The gender was only used for adjustment in analysis of overall population.

^c^ High physical activity was defined as muscle strengthening exercise, such as push-ups, sit-ups, dumbbell curls for one week for at least 3 days.

^d^ Model 2 was calculated by logistic regression analyses after matching on the propensity score.

## Discussion

Our study showed that low HGS was significantly associated with LBP. We analyzed 5607 participants, and it was a meaningful indicator in both men and women when confounding factors were not considered. However, when confounding factors were considered, significant results were obtained for women aged over 50 years with low physical activity. These results were similar when analyzed in matched groups by propensity score. Thus, our study indicated the relationship between low HGS and LBP in Korean women aged 50 years or older with low physical activity rather than in Korean men within the same age group.

HGS is a reliable measurement technique for the assessment of maximal grip strength force and evaluation of overall muscle strength, nutritional status, muscle mass, and walking performance [10][12]. HGS is an important factor in diagnosing sarcopenia and is used to define low muscle strength [16]. Low HGS is of prognostic value in forecasting the future clinical outcome of patients with low muscle mass and poor mobility (e.g., mortality, disability, resource utilization) [17][18]. It was defined as 30 kgf or less for men and 20 kgf or less for women according to the European Working Group on Sarcopenia in Older People criteria and 26 kgf for men and 18 kgf for women according to the Asian Working Group for Sarcopenia criteria [16][19]. However, both of these criteria are not Korean standards. We used recently determined Korean cut-off values of 28.6 kgf and 16.4 kgf for low HGS in Korean men and women, respectively [11].

Some studies have reported the relationship between low muscle strength and LBP [7][8][9]. However, measurement of back muscle strength using a standardized method not only is difficult but also requires expensive tools. Measuring the back muscle mass is also difficult because of similar reasons. Most studies have shown that muscle-strengthening exercises, including core exercises, reduce LBP [4][5][6]. In particular, it has been reported that exercise for 2 days or more per week on nonconsecutive days significantly reduces LBP [4][5]. The relation of low skeletal muscle mass of the extremities to other musculoskeletal pain has been reported [9][20]. These reports have indicated that low muscle mass of the extremities is associated with knee pain [20] and neck or shoulder pain [9], but is not related to LBP. To our knowledge, only one study analyzed the direct relationship between trunk muscle strength and LBP and showed the association between isometric and isokinetic extensor weakness and LBP [7]. Although its study design is good in that trunk muscle strength was measured, the number of subjects was too small to provide a conclusive result for the general population. In our study, we compared LBP with HGS; we could not directly measure back muscle strength but could simply replace the muscle strength of the whole body. It is possible that our study may have more inaccuracies than would be observed by direct objective measurements. Nonetheless, it is derived from a representative dataset sampled from the Korean general population and may therefore have broad applicability.

In Japan, the relationship between sarcopenia and LBP has recently been reported [8]. In this study, the pre-sarcopenia and sarcopenia groups had higher visual analog scale pain and ODI scores. Although there is an advantage in recognizing the relationship between the three diagnostic factors for sarcopenia (skeletal muscle mass, HGS, and gait speed) and LBP, there were only 12 subjects in the sarcopenia group, which is a major disadvantage. As we used the KNHANES database, we analyzed a larger population than that in the previous study. In addition, multiple logistic regression and propensity score matching were used to control for sociodemographic and environmental factors and for comorbidities affecting LBP. Before controlling for confounding factors, low HGS with low physical activity was found to affect both men and women. However, after controlling for confounding factors, the association was observed only in women with low physical activity. The ORs in the two models were somewhat different but were statistically significant for women with low physical activity. However, no previous study has explored why significant results were shown only in women. The difference in cut-off values between men and women is thought to explain our results. However, the cut-off value for integrating men and women has not yet been determined. For this reason, we analyzed HGS according to men and women, and the result was as mentioned above.

When providing treatment, physicians should be aware that low muscle strength is a risk factor for LBP. In general, when patients have back pain, physicians treat only organic spinal problems. Patients who do not respond to orthopedic care should also undergo an HGS test. The HGS test can be useful for screening low muscle strength. If low HGS is confirmed using a dynamometer, patients might benefit from the addition of physical activities, including aerobic and core exercises, to treatments for organic spinal problems and chronic LBP. Decreasing fear and increasing exercise will help reduce LBP [21]. Therefore, comprehensive treatment of not only anatomical but also physical aspects of chronic LBP will be helpful for excellent clinical outcomes. Further studies are required to test whether it is possible to reduce the incidence of LBP with exercise.

To the best of our knowledge, this is the first study investigating sex differences in the association between low muscle strength and LBP in a nationwide Korean representative sample of population aged 50 years and older. The greatest strength of our study was to increase the external validity of our findings using the KNHANES data, which provides representative samples of the Korean general population. However, some limitations should also be noted. First, our study had a cross-sectional design with respect to assessing the KNHANES data. Therefore, we cannot analyze the causal relationships between low muscle strength and LBP and can only provide ORs. However, the survey datasets were designed to minimize sampling errors, and the association between low HGS and LBP can be considered highly representative. Second, the surveys used in this study did not evaluate the degree of LBP, which is usually conducted using a visual analog pain scale. Third, the relationship between low muscle strength and LBP may be dependent on ethnicity. Hence, our results can be generalized to the Korean population only. Fourth, muscle strength was measured using a handgrip dynamometer. The most accurate way to measure muscle strength that is directly associated with LBP is to use a dynamometer for trunk muscle strength. However, this instrumentation is very expensive and difficult to use in the general population. In addition, measurement of HGS may be a substitute for the assessment of both general muscle strength and nutritional status [10][12]. Fifth, the definition of LBP was uncertain. We only defined the presence of LBP according to LBP duration. We could not consider anatomic problems such as disc herniation or trauma because of the limitations of the KNHANES dataset. However, LBP has several anatomical problems, but it is difficult to pinpoint the cause of LBP because there are usually overlapping anatomical problems. Therefore, it is also advantageous to include all types of LBP in this study.

## Conclusions

Our study showed the relationship between HGS and LBP in the Korean general population. Low HGS in women aged over 50 years with low physical activity was significantly associated with the presence of LBP. Thus, physicians should be aware that low HGS is significantly associated with the presence of LBP and must therefore counsel female patients aged over 50 years with low HGS to increase their level of physical activities to improve LBP.
